# Differential Associations for Salivary Sodium, Potassium, Calcium, and Phosphate Levels with Carotid Intima Media Thickness, Heart Rate, and Arterial Stiffness

**DOI:** 10.1155/2018/3152146

**Published:** 2018-12-16

**Authors:** Carlos Labat, Silke Thul, John Pirault, Mohamed Temmar, Simon N. Thornton, Athanase Benetos, Magnus Bäck

**Affiliations:** ^1^INSERM U1116, Université de Lorraine, Nancy, France; ^2^Translational Cardiology, Karolinska Institutet, Stockholm, Sweden; ^3^Telomere Cardiology Center, Ghardaia, Algeria; ^4^Centre Hospitalier Universitaire, Nancy, France; ^5^Theme Heart and Vessels, Division of Valvular and Coronary Diseases, Karolinska University Hospital, Stockholm, Sweden

## Abstract

Salivary biomarkers may offer a noninvasive and easy sampling alternative in cardiovascular risk evaluation. The aim of the present study was to establish associations of salivary potassium, sodium, calcium, and phosphate levels with the cardiovascular phenotype determined by carotid ultrasound and carotid-femoral pulse wave velocity and to identify possible covariates for these associations. *N* = 241 samples of nonstimulated whole buccal saliva were obtained from subjects with (*n* = 143; 59%) or without (*n* = 98; 41%) hypertension. The potassium concentrations were 10-fold higher in saliva compared with plasma, whereas sodium concentrations exhibited the reverse relation between saliva and blood. There were no significant correlations between the levels of sodium, potassium, or calcium in saliva and plasma. All salivary electrolytes, except sodium, were significantly associated with age. In age-adjusted analyses, salivary potassium was significantly associated with carotid artery intima media thickness (cIMT) and carotid-femoral pulse wave velocity, and these associations were at the limit of significance in multivariate analyses including prevalent cardiovascular disease and risk factors. Body mass index was a significant confounder for salivary potassium. Salivary phosphate was significantly associated with cIMT in the multivariate analysis. Salivary potassium, calcium, and phosphate levels were significantly associated with heart rate in the univariate age-adjusted as well as in two different multivariate models, whereas no significant associations between sodium and heart rate were observed. In conclusion, the differential association of salivary electrolytes with cardiovascular phenotypes indicates that these electrolytes should be further studied for their predictive value as noninvasive biomarkers for cardiovascular risk evaluation.

## 1. Introduction

Saliva is gaining renewed interest for its diagnostic value. The simple and noninvasive sampling procedure for saliva, together with the development of easy-to-use point of care analysis methods, may change the landscape of medical diagnostics and facilitate patient evaluation by telemedicine. The measurement of C-reactive protein (CRP) in saliva is one such example. Since salivary CRP correlates strongly with circulating levels [1], the use of venipuncture can be avoided, hence permitting the patients to sample saliva themselves for infectious and inflammatory disease diagnosis.

Low levels of CRP, measured by high-sensitivity CRP assays, are indicative of low-grade chronic inflammation, which can be associated with an increased cardiovascular risk [2]. Importantly, such CRP alteration within the lower normal range can also be detected in saliva. Consequently, salivary high-sensitivity CRP predicts subclinical atherosclerosis, carotid artery intima media thickness (cIMT), and increased vascular stiffness [1]. Likewise, salivary CRP can be used in rheumatic diseases to monitor inflammatory activity and the effect of anti-inflammatory treatments [3]. In addition to CRP, there are also a number of other salivary biomarkers that have been associated with cIMT [1, 4] and also with age [5], diabetes [6], and chronic kidney disease (CKD) [7, 8].

The sodium to potassium ratio in stimulated saliva has been used historically as index of the total mineralocorticoid effect, for example, in hypertension [9]. Saliva from subjects with diabetes exhibits significantly higher potassium concentrations compared with controls, whereas salivary sodium, calcium, and phosphate are similar between diabetics and non-diabetics [6]. Likewise, salivary levels of potassium and chloride decrease after hemodialysis in end-stage CKD, whereas calcium and sodium levels remain unchanged. These studies hence suggested a specific regulation of salivary ion concentrations in different diseases. Sodium, potassium, and other electrolytes in plasma have been extensively studied in relation to cardiovascular diseases and may reflect, for example, dietary intake [10], renal excretion [7], and activation of the renin-angiotensin and aldosterone systems [9, 11]. However, the predictive value of different salivary electrolytes for cardiovascular phenotypes has remained unexplored.

The aim of the present study was therefore to establish associations of salivary potassium, sodium, calcium, and phosphate levels with the cardiovascular phenotype determined by carotid ultrasound and carotid-femoral pulse wave velocity and to identify possible covariates for these associations.

## 2. Methods

### 2.1. Study Population

Subjects were participants in the ERA (Etude de la Rigidité Artérielle) Study, a prospective study of carotid-femoral pulse wave velocity (cfPWV), initiated in 1992–1993 as previously described [1, 4, 12, 13]. All ERA Study participants who participated in the first follow-up visit in 1998–1999 were invited to participate in a second follow-up visit in 2008. In response to this invitation, 271 subjects were examined at the Centre d'Investigations Préventives et Cliniques (the IPC Center, 6/14, rue La Pérouse, 75116 Paris, France), and saliva samples were obtained from 259 of these subjects. For the present study, sufficient volume remained for electrolyte measurements in samples from 241 subjects. The study protocol was approved by the local ethics committee (Comité d'Ethique du Centre Hospitalier Universitaire de Cochin) and written informed consent was obtained from all study participants.

### 2.2. Clinical Investigations

Ultrasound examinations were performed using the Aloka SSD-650, with a transducer frequency of 7.5 MHz as previously described [14]. Acquisition, processing, and storage of B-mode images were computer assisted using the M'ATHS software (Metris, France). The protocol involved scanning of the common carotid arteries, the carotid bifurcations, and the origin (first 2 cm) of the internal carotid arteries. At the time of the examination, the near and far walls of these arterial segments were scanned longitudinally and transversally to assess the presence of plaques. The presence of plaques was defined as localized echo-structures encroaching into the vessel lumen for which the distance between the media-adventitia interface and the internal side of the lesion was 1 mm. For intima-media thickness and lumen diameter measurements, near and far walls of the right and the left common carotid arteries, 2 to 3 cm proximal to bifurcation were imaged. In patients with carotid artery plaques, intima-media thickness measurements were realized in plaque-free segments of the common carotid arteries. Details of the methodology used have been previously described [13, 15].

Carotid-femoral pulse wave velocity (cfPWV) was measured at a constant room temperature of 19°C to 21°C and calculated using Complior (Colson, Garges les Genosse, France) as previously described [12]. Briefly, two pressure waves were recorded transcutaneously at the base of the neck for the right common carotid artery and over the right femoral artery. CfPWV was determined as the foot-to-foot velocity. Pulse transit time was determined as the average of 10 consecutive beats. The distance traveled by the pulse wave was measured over the body surface as the distance between the 2 recording sites.

Pulse pressure (PP) was calculated from supine blood pressure measurements using a manual sphygmomanometer. After a 10-minute rest period, blood pressure was measured 3 times, and the average of the last 2 measurements was used for statistical analyses.

### 2.3. Saliva Collection and Preparation

Unstimulated whole buccal saliva was collected from subjects as previously described [1, 4, 5, 16], at the time of the second follow-up visit in 2008. Briefly, saliva was collected during 3 minutes after an overnight fast and without prior oral hygiene measures. Subjects were not informed to abstain from smoking before saliva collection. Saliva samples were immediately frozen at −80°C and stored for less than 3 months before biochemical analysis. At thawing, the collected saliva volume was measured, followed by centrifugation of the sample (4000 rpm/10 min/4°C), and prepared into aliquots for each analysis.

### 2.4. Biochemical Measurements

Sodium, potassium, and calcium were measured in plasma and saliva and phosphate in saliva. Total plasma cholesterol, high- and low-density lipoprotein (HDL and LDL, respectively), cholesterol, and triglycerides were also measured. The salivary electrolytes were measured in centrifuged saliva samples on a Cobas 8000 c701 chemistry analyzer (Roche Diagnostics) at the Karolinska University Laboratory, Karolinska University Hospital, Solna, Sweden.

### 2.5. Statistics

Clinical parameters, plasma, and salivary measures are expressed as either percent or mean ± SD or median (interquartile range) for normally and nonnormally distributed data, respectively. Statistically significant differences were determined using either a Student's *t*-test (normally distributed data) or Wilcoxon signed-rank test (nonnormally distributed data) for continuous variable. A chi-square test was used for categorical data. Correlations between the salivary and plasma concentrations and correlations with age were established by Person correlation. A multiple linear regression was performed to evaluate salivary biomarkers as predictors of the clinical parameters monitored and to establish the correlation coefficient for each association. In the multivariate analyses, correlations were adjusted for age, for age plus all cardiovascular phenotypes (model 1), and for age, all cardiovascular phenotypes plus prevalent cardiovascular disease and risk factors (model 2). A *P* value < 0.05 was considered significant. All analyses were performed using the NCSS 2000 statistical software package (NCSS, LLC, Kaysville, Utah, USA).

## 3. Results

### 3.1. Baseline Characteristics and Stratification according to Prevalent Hypertension

The baseline characteristics of the *n* = 241 subjects are shown in Table 1. Subjects with hypertension were significantly older and had significantly higher prevalence of other cardiovascular diseases, more signs of subclinical atherosclerosis on carotid ultrasound, higher BMI, and increased cfPWV. In the plasma analysis, hypertensive subjects had higher levels of glucose, LDL cholesterol, and TGs and lower levels of HDL. Calcium levels in both plasma and saliva were higher in hypertensive subjects. Salivary levels, but not plasma levels, of potassium were significantly higher in subjects with hypertension (Table 1). Stratification based on sex did not reveal any significant differences in terms of the measured circulatory and salivary electrolytes (Supplementary Table 1).

### 3.2. Substantial Concentration Differences and Lack of Correlations between Saliva and Plasma Electrolytes

**Figure 1:**
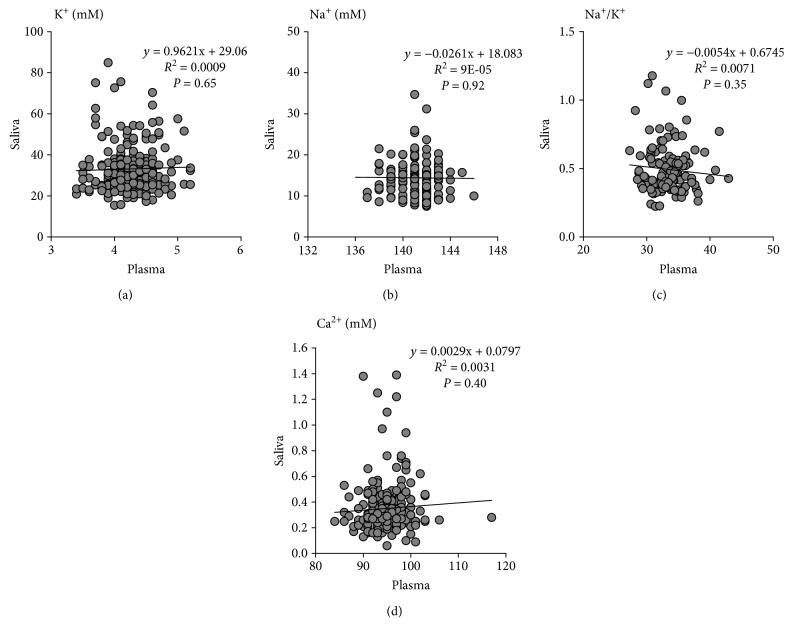
Comparison between saliva and plasma electrolytes. The potassium concentrations were 10-fold higher in saliva compared with plasma (a), whereas sodium concentrations exhibited the reverse relation between saliva and blood (b). Consequently, the sodium to potassium ratio was inversed in the saliva, being 100-fold lower compared with plasma (c). There were no significant associations between saliva and plasma for potassium concentrations (a), sodium concentrations (b), or the sodium to potassium ratio (c). The salivary calcium levels were more than a 100-fold lower compared with plasma (d). There were no significant associations between saliva and plasma calcium concentrations (d). Pearson correlation coefficients and *P* values are indicated in each panel.

The potassium concentrations were 10-fold higher in saliva compared with plasma, whereas sodium concentrations exhibited the reverse relation between saliva and blood (Figures 1(a) and 1(b)). Consequently, the sodium to potassium ratio was inversed in the saliva, being 100-fold lower compared with plasma (Figure 1(c)). There were no significant correlations between saliva and plasma for potassium, sodium, or the sodium to potassium ratio (Figure 1). The salivary calcium levels were low, more than a 100-fold lower compared with plasma (Table 1), and no significant correlation between the two compartments was observed for calcium concentrations (Figure 1(d)). Salivary phosphate was 7.47 ± 3.51 mmol/L (Table 1), which was higher compared with the reference levels in plasma (1.12–1.45 mmol/L).

### 3.3. Salivary Electrolytes Are Increased with Age

**Figure 2:**
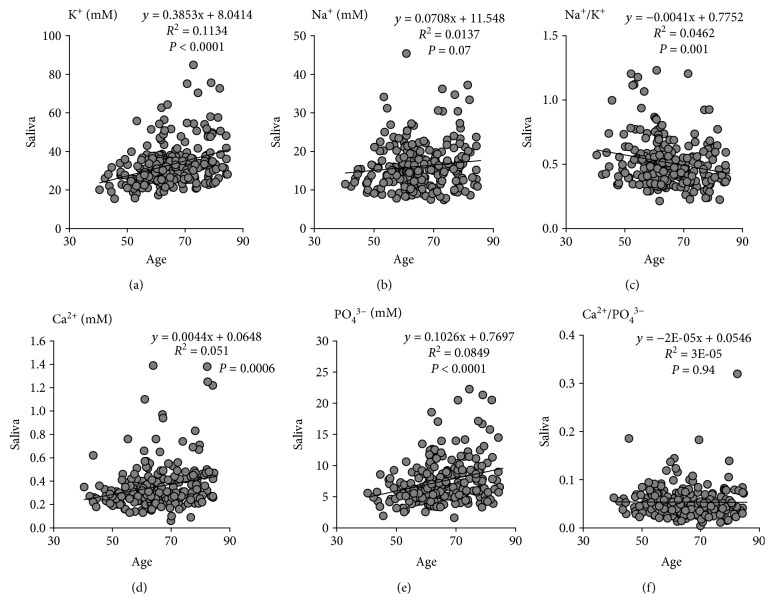
Salivary electrolytes are increased with age. There was a significant correlation between salivary potassium concentrations (a) but not sodium concentrations (b) with age. The sodium to potassium ratio (c) was inversely correlated with age. Calcium concentrations (d) and phosphate concentrations (e) but not the calcium to phosphate ratio were significantly correlated with age. Pearson correlation coefficients and *P* values are indicated in each panel.

Potassium exhibited a strong and positive association with age (Figure 2(a)), whereas the correlation between sodium and age was weaker and did not reach statistical significance (Figure 2(b)). The sodium to potassium ratio declined significantly with age (Figure 2(c)). Calcium and phosphate concentrations in saliva were both significantly increased with age (Figures 2(d) and 2(e)), whereas the calcium to phosphate ratio remained unchanged (Figure 2(f)).

### 3.4. Salivary Electrolytes Are Differentially Associated with the Cardiovascular Phenotype

Plasma levels of sodium and potassium were not significantly associated with any of the cardiovascular phenotypes examined in the present study: PP, cIMT, carotid plaque, cfPWV or HR (Supplementary Table 2). Plasma calcium levels were significantly associated with prevalent hypertension, metabolic syndrome, and BMI but not with any of the other parameters (Supplementary Table 2). We next performed association analyses to determine the predictive value of the measured salivary electrolytes for each of these cardiovascular phenotypes.

#### 3.4.1. Sodium

In univariate analysis, salivary sodium was significantly associated with PP, cIMT, and cfPWV (Table 2). PP and IMT remained significantly associated with sodium in the univariate analysis after adjustment for age (Table 2). However, in the multiple regression model including all cardiovascular phenotypes, these associations did not attain statistical significance (Table 2; model 1). Likewise, adding also prevalent cardiovascular disease and risk factors to the model did not reveal any predictive value of salivary sodium for any of the included covariates (Table 2; model 2).

#### 3.4.2. Potassium

Salivary potassium was significantly associated with PP, HR, cfPWV, plaque, and cIMT in the univariate analysis and with HR, cfPWV, and cIMT after age adjustment. These parameters remained significant in the multivariate analysis, albeit with a trend association for cIMT (Table 2; model 1). With further adjustment for prevalent cardiovascular disease and risk factors, the associations of salivary potassium with cIMT and cfPWV were at the limit of significance, whereas the association with HR remained significant (Table 2; model 2). In the latter analysis, BMI was revealed to be a significant covariate for salivary potassium levels (Table 2; model 2).

#### 3.4.3. Calcium

Salivary calcium was significantly associated with PP, HR, cfPWV, IMT, and plaque in the unadjusted univariate analysis but only with HR after age adjustment. Likewise, the association between salivary calcium and HR remained significant in both multivariate analysis models (Table 3; models 1 and 2).

#### 3.4.4. Phosphate

Similar to the results obtained for calcium, univariate analysis for salivary phosphate revealed significant associations with PP, HR, cfPWV, cIMT, and carotid plaque in the unadjusted analysis. The association for salivary phosphate with HR and cfPWV remained significant after adjustment for age. The multiple regression model identified that salivary phosphate levels predicted HR and cIMT in this cohort, which both remained significant also in the mode including prevalent cardiovascular disease and risk factors (Table 3; models 1 and 2).

### 3.5. Salivary Electrolytes in relation to Antihypertensive Treatments

Subjects taking beta-blockers exhibited significantly higher salivary levels of sodium, potassium, and calcium, whereas phosphate levels were not different between users and nonusers (Table 4). Phosphate levels were however increased in subjects treated with calcium channel blockers, which also exhibited higher potassium levels. In contrast, users of angiotensin-modifying drugs (ACE inhibitors or AT1 receptor blockers) presented similar salivary ion levels as nonusers (Table 4). There was however a significant difference in terms of age between users and nonusers in all the medication groups studied (Table 4). Nevertheless, a multivariate analysis including age and all four groups of medications conformed significant associations for the salivary levels of potassium (coefficient = 4.43 ± 1.70; *R* ^2^ = 2.5%; *P* = 0.01) and sodium (coefficient = 2.83 ± 0.93; *R* ^2^ = 3.8%; *P* = 0.003) with the use of beta-blockers. The association between salivary phosphate and the use of calcium channel blockers did not reach statistical significance in the age-adjusted multivariate model (coefficient = 1.25 ± 0.64; *R* ^2^ = 2.5%; *P* = 0.051). Likewise, the use of either diuretics or angiotensin-modifying drugs was not significantly associated with any of the salivary ions in the multivariate model.

## 4. Discussion

Three major observations emerge from the present study. First, salivary levels of sodium, potassium, calcium, and phosphate were different from the plasma levels of these ions. Second, salivary ion concentrations increased with age and exhibited significant associations with cardiovascular phenotypes. Third, in multivariate analysis adjusted for age and including prevalent cardiovascular disease and risk factors, we found HR to be a major determinant for salivary ion levels and we identified also salivary phosphate levels as an independent predictor of cIMT.

Previous studies compared plasma and saliva levels of different ions [11, 17]. In the present study, the salivary levels of sodium, potassium, and calcium were not correlated with their corresponding plasma concentrations. Interestingly, the Na/K ratio was inversed, with higher potassium and lower sodium in saliva as compared with plasma. These observations confirm previous observations [11] and may reflect the active sodium resorption in salivary glands by a Na-K-ATPase pump, which is responsive to aldosterone [10]. In contrast, potassium is actively released to the saliva by, for example, nerve stimulation. This argues in favor of a specific diagnostic and predictive value for salivary sodium and potassium.

Calcium concentrations are low in the saliva [6], and we show in the present study substantially lower calcium in saliva compared with plasma. In contrast, phosphate was readily detectable at levels higher than their physiological plasma levels. In fact, the phosphate levels were far above the levels where phosphate is considered to precipitate and cause calcification. It is therefore interesting that abundant levels of calcification inhibitors can be found in the saliva [18, 19], but their relation to phosphate levels remains to be established. Indeed, drivers of calcification, such as hyperparathyroidism, increase salivary phosphate levels [8].

Whereas salivary levels were not significantly different between men and women in the present study, there was a general pattern of significant associations of all salivary measures with age. This is consistent with previous studies establishing age as a general confounder for salivary ions [17] and other biomarkers in saliva [5] and substantial alterations in salivary composition in elderly subjects [20]. However, not all ions exhibited similar degrees of increase with age. For example, a more important rise in salivary potassium with age was observed compared with nonsignificant changes in sodium, resulting in a decreased sodium to potassium ratio with age. In contrast, the age-related rise in salivary calcium and phosphate were similar resulting in a constant calcium to phosphate ratio over the age-interval studies. To compensate for these age-related effects, all our subsequent analyses in the present study were adjusted for age.

Carotid artery ultrasound examination can be used to determine the presence of atherosclerotic plaques and/or arterial thickening (cIMT) and may be considered in cardiovascular risk prediction [21]. Previous studies have shown that salivary levels of inflammatory markers, *e.g.*, CRP, matrix metalloproteinase (MMP)-9 [1], and the resolvin to leukotriene ratio [4], are significantly associated with cIMT. In the present study, we identified significant associations for all measured salivary electrolytes with carotid artery atherosclerotic plaques and/or cIMT. For sodium and potassium, the associations with cIMT remained significant in the age-adjusted analyses and were at the limit of significance in the two different multivariate models.

The association between phosphate and cIMT remained significant in all models. In patients with CKD, a significant correlation between serum phosphate and cIMT has been reported, independent of other cardiovascular risk factors [22]. There is also an association of teriparatide treatment (which increases the urinary excretion of phosphate) with a reduction in cIMT and increased bone density [23]. These studies point to increased phosphate as a risk factor for early atherosclerosis. Interestingly, salivary phosphate levels are also increased in CKD patients despite hemodialysis and phosphate binder treatment [24] and may represent a more sensitive measure of a disturbed phosphate balance with implications for cardiovascular disease. The results of the present study for the first time raise the notion of salivary phosphate levels as a predictor of subclinical cardiovascular disease in the absence of kidney disease.

The final cardiovascular phenotype assessed was arterial stiffness by means of cfPWV, which previously has been associated with salivary levels of the inflammatory lipid mediator leukotriene B_4_ [1]. Salivary potassium and phosphate were associated with cfPWV in the age-adjusted univariate analysis, further reinforcing the value of these salivary biomarkers for cardiovascular disease. The observed association between cfPWV and salivary potassium remained significant when all cardiovascular phenotypes were taken into consideration but was at the limit of significance when prevalent cardiovascular disease and risk factors were included in the model. These observations indicate that other risk factors, mainly BMI, may be a significant confounder for the association of salivary potassium with cardiovascular phenotypes, which warrant further exploration.

There are several possible mechanisms involved in the observed associations. Salivary electrolytes may be related to dietary factors, such as reduced salt intake, which lowers salivary sodium and increases salivary potassium, as a consequence of a lowered sodium to potassium ratio. Likewise, the sodium to potassium ratio may reflect mineralocorticoid activity [9]. It should however be pointed out that stimulated saliva was used in previous studies [11] and that the collection of unstimulated saliva used in the present study may be less regulated by aldosterone [25]. Importantly, however, potassium and not sodium was predictive of the cardiovascular phenotypes studied in this cohort, further reinforcing that the observed associations were specific for potassium. Another modulator of salivary ions is the sympathetic nervous system [25, 26]. We note especially how potassium, calcium, and phosphate levels were all significantly associated with HR in the univariate age-adjusted as well as the two different multivariate models, whereas no significant associations between sodium and HR were observed. However, also hydration status may affect both HR and salivary composition [27], which also should be taken into consideration in the context of salivary biomarkers. Saliva osmolarity however reflects age-dependent effects on hydration station, and it should be pointed out that the significant associations between salivary ions and cardiovascular phenotypes were observed in age-adjusted analyses.

Subjects using beta-blockers exhibited significantly higher levels of salivary sodium and potassium compared with nonusers and this difference remained in the multivariate analysis. Both *β*1 and *β*2 adrenoreceptors have been shown to regulate salivary gland sodium reabsorption and potassium secretion in experimental models [26], suggesting that antihypertensive use of beta-blockers may directly alter salivary ions. In contrast, the use of either calcium blockers or diuretics was not associated with the salivary electrolyte levels in the multivariate analysis. Likewise, the lack of alterations of salivary electrolytes by angiotensin-modifying medications (ACE inhibitors and AT1 receptor blockers) is consistent with previous observations in hypertensive subjects [10].

The strengths of the present study include the careful cardiovascular phenotyping and monitoring of prevalent cardiovascular disease and risk factors, as well as treatments, allowing the possibility to establish the associations determined by different salivary electrolytes. There are however some limitations, which should be acknowledged. First, we lacked information on dietary intake, which may have contributed to the concentrations measured. Also, different sources of water were not monitored and may have interfered with the levels detected. Although we report for the first time associations between salivary ions and HR, more precise measures, such as heart rate variability, would be needed to make a firm conclusion on the relation between salivary electrolytes and sympathetic tone. Although hypertension was common, the present cohort was otherwise relatively healthy, and the applicability of the observed associations to other populations remains to be established. Other prevalent comorbidities were not monitored, and it is unclear how, for example, periodontal status may have affected the observed associations. Information on the use of other medications as well as the doses used was also not collected. Finally, given the major impact of age on salivary electrolytes, further studies are needed to establish how to distinguish normal and pathological cardiovascular aging using salivary biomarkers.

## 5. Conclusion

In summary, salivary potassium was increased in hypertension and associated with vascular stiffness and cIMT. However, BMI was a significant confounder and the associations between salivary potassium and cardiovascular phenotypes were at the limit of significance in the fully adjusted multivariate model. Furthermore, the present study identifies salivary phosphate as an independent predictor of cIMT and the association of several salivary electrolytes with HR. In conclusion, the differential association of salivary electrolytes with cardiovascular phenotypes indicates that these electrolytes should be further studied for their predictive value as noninvasive biomarkers for determining cardiovascular risk.

## Figures and Tables

**Table 1:** Cohort characteristics stratified according to hypertension.

	All	No hypertension	Hypertension
*N* (%)	241	98 (41%)	143 (59%)
Age (years)	65 ± 10	61 ± 10	69 ± 9^∗∗∗^
Women	30%	33%	29%
BMI (kg/m^2^)	26.5 ± 4.0	25.0 ± 3.3	27.6 ± 4.1^∗∗∗^
Metabolic syndrome	53%	27%	71%^∗∗∗^
Diabetes	1.7%	0.0%	2.8%
Antihypertensive treatment	54%	0%	91%^∗∗∗^
CV disease	12%	4%	17%
*Cardiovascular phenotypes*			
PP (mmHg)	55 ± 16	46 ± 12	61 ± 16^∗∗∗^
HR (min^−1^)	69 ± 11	68 ± 9	70 ± 11
Carotid IMT (mm)	0.75 ± 0.11	0.69 ± 0.09	0.79 ± 0.11^∗∗∗^
Carotid plaque (yes)	54%	34%	68%^∗∗∗^
cfPWV (m/s)	12.9 ± 3.7	11.3 ± 2.7	14.0 ± 3.9^∗∗∗^
*Saliva parameters*			
Sodium (mM)	15.2 (12.2–19.3)	14.5 (11.5–18.6)	15.4 (12.8–19.4)
Potassium (mM)	31.2 (25.5–36.8)	29.2 (25.1–34.7)	32.4 (26.0–38.5)^∗∗^
Phosphate (mM)	6.61 (5.16–8.99)	6.33 (5.10–8.78)	6.80 (5.24–9.18)
Calcium (mM)	0.31 (0.25–0.40)	0.28 (0.23–0.37)	0.31 (0.26–0.42)^∗^
Sodium/potassium ratio	0.48 (0.39–0.58)	0.48 (0.38–0.60)	0.47 (0.39–0.56)
Calcium/phosphate ratio	0.047 (0.032–0.067)	0.046 (0.032–0.063)	0.048 (0.032–0.072)
*Plasma parameters*			
Potassium (mM)	4.2 (4.0–4.5)	4.3 (4.1–4.5)	4.2 (4.0–4.5)
Sodium (mM)	140 (140–142)	141 (140–142)	142 (140–142)
Calcium (mM)	95 (93–97)	94 (92–96)	96 (93–98)^∗∗∗^
Sodium/potassium ratio	33.5 (31.6–35.0)	33.1 (31.7–34.9)	33.8 (31.2–35.3)
Total cholesterol (g/L)	2.14 (1.93–2.39)	2.15 (1.93–2.39)	2.10 (1.90–2.40)
LDL cholesterol (g/L)	1.45 (1.25–1.65)	1.50 (1.30–1.66)	1.39 (1.20–1.59)^∗^
HDL cholesterol (g/L)	0.49 (0.41–0.59)	0.52 (0.44–0.63)	0.47 (0.39–0.57)^∗∗^
Triglycerides (g/L)	0.97 (0.74–1.26)	0.90 (0.72–1.08)	1.08 (0.81–1.55)^∗∗^
Glucose (g/L)	0.99 (0.94–1.06)	0.97 (0.93–1.03)	1.01 (0.95–1.08)^∗∗^

Data are expressed as either mean ± SD or median (interquartile range) for normally and nonnormally distributed data, respectively. Statistically significant differences were determined using either a Student's *t*-test (normally distributed data) or Wilcoxon signed-rank test (nonnormally distributed data) for continuous variable. A chi-square test was used for categorical data. ^∗^ *P* < 0.05; ^∗∗^ *P* < 0.01; ^∗∗∗^ *P* < 0.001 vs. no hypertension.

**Table 2:** Associations for salivary sodium and potassium with age, sex, and cardiometabolic phenotypes.

	Sodium			Potassium		
	Regr coeff ± SE	*R* ^2^	Prob Level	Regr coeff ± SE	*R* ^2^	Prob level
*Univariate*						
Age (years)	0.070 ± 0.040	1.4%	0.07	0.39 ± 0.07	11.3%	<0.0001
Women (yes)	−0.46 ± 0.85	0.1%	0.58	−1.07 ± 1.61	0.2%	0.51
PP (mmHg)	0.073 ± 0.023	4.1%	0.002	0.18 ± 0.04	7.0%	<0.0001
HR (min^−1^)	0.057 ± 0.037	1.0%	0.13	0.20 ± 0.07	3.5%	0.004
cIMT (mm)	10.12 ± 3.41	3.7%	0.003	29.8 ± 6.3	8.8%	<0.0001
Carotid plaque (yes)	0.385 ± 0.794	0.1%	0.63	5.12 ± 1.4	5.4%	0.0004
cfPWV (m/s)	0.247 ± 0.104	2.4%	0.02	1.01 ± 0.19	11.2%	<0.0001
Hypertension (yes)	1.245 ± 0.786	1.1%	0.11	4.72 ± 1.46	4.3%	0.001
CV disease (yes)	1.420 ± 1.215	0.6%	0.24	1.73 ± 2.31	0.2%	0.45
Diabetes (yes)	7.505 ± 4.199	1.4%	0.08	3.68 ± 6.55	0.1%	0.57
Metabolic syndrome (yes)	0.599 ± 0.783	0.2%	0.44	4.28 ± 1.46	3.6%	0.004
BMI (kg/m^2^)	0.213 ± 0.099	2.0%	0.03	0.55 ± 0.18	3.7%	0.003

*Age adjusted*						
Women	−0.72 ± 0.85	0.3%	0.40	−2.36 ± 1.53	0.9%	0.12
PP (mmHg)	0.072 ± 0.028	2.7%	0.01	0.078 ± 0.05	0.9%	0.13
HR (min^−1^)	0.054 ± 0.037	0.9%	0.15	0.18 ± 0.07	2.9%	0.006
cIMT (mm)	9.75 ± 4.16	2.3%	0.02	15.72 ± 7.46	1.7%	0.04
Carotid plaque (yes)	−0.22 ± 0.87	0.0%	0.80	2.42 ± 1..50	1.0%	0.11
cfPWV (m/s)	0.20 ± 0.12	1.2%	0.10	0.66 ± 0.21	3.4%	0.003
Hypertension (yes)	0.81 ± 0.86	0.4%	0.35	1.93 ± 1.54	0.6%	0.21
CV disease (yes)	1.16 ± 1.22	0.4%	0.34	0.22 ± 2.20	0.0%	0.92
Diabetes (yes)	7.74 ± 4.18	1.5%	0.07	2.70 ± 6.19	0.1%	0.66
Metabolic syndrome (yes)	0.26 ± 0.81	0.1%	0.75	2.45 ± 1.44	1.1%	0.09
BMI (kg/m^2^)	0.21 ± 0.10	2.0%	0.03	0.54 ± 0.17	3.6%	0.002

*Model 1*						
Age (years)	−0.0.30 ± 0.05	0.1%	0.56	0.20 ± 0.09	1.7%	0.03
PP (mmHg)	0.057 ± 0.029	1.6%	0.053	—	—	0.46
HR (min^−1^)	—	—	0.15	0.15 ± 0.07	1.8%	0.02
cIMT (mm)	7.26 ± 4.32	1.2%	0.09	13.26 ± 7.40	1.1%	0.07
Carotid plaque (yes)	—	—	0.17	—	—	0.41
cfPWV (m/s)	—	—	0.44	0.48 ± 0.22	1.7%	0.03
Model		5.2%			18.4%	

*Model 2*						
Age (years)	−0.030 ± 0.05	0.1%	0.56	0.20 ± 0.09	1.8%	0.02
PP (mmHg)	0.057 ± 0.029	1.6%	0.053	—	—	0.79
HR (min^−1^)	—	—	0.15	0.14 ± 0.06	1.7%	0.03
cIMT (mm)	7.26 ± 4.32	1.2%	0.09	14.65 ± 7.45	1.4%	0.05
Carotid plaque (yes)	—	—	0.17	—	—	0.57
cfPWV (m/s)	—	—	0.44	0.35 ± 0.21	1.0%	0.10
Hypertesion (yes)	—	—	0.65	—	—	0.54
CV disease (yes)	—	—	0.35	—	—	0.59
Diabetes (yes)	—	—	0.11	—	—	0.79
Metabolic syndrome (yes)	—	—	0.56	—	—	0.63
BMI (kg/m^2^)	—	—	0.2	0.41 ± 0.17	1.8%	0.02
Model		5.2%			20.8%	

Regr coeff: regression coefficient; SE: standard error; prob level: probability level.

**Table 3:** Associations for salivary calcium and phosphate with age, sex, and cardiometabolic phenotypes.

Dependent	Calcium			Phosphate		
	Regr coeff ± SE	*R* ^2^	Prob level	Regr coeff ± SE	*R* ^2^	Prob level
*Univariate*						
Age (years)	0.0044 ± 0.00112	5.1%	0.0005	0.10 ± 0.02	8.5%	<0.0001
Women (yes)	0.0073 ± 0.0279	0.0%	0.79	−0.17 ± 0.51	0.0%	0.73
PP (mmHg)	0.0020 ± 0.0008	2.6%	0.01	0.037 ± 0.014	2.9%	0.01
HR (min^−1^)	0.0035 ± 0.0012	3.5%	0.004	0.052 ± 0.022	2.2%	0.02
cIMT (mm)	0.33 ± 0.11	3.9%	0.003	7.38 ± 2.01	5.6%	0.0003
Carotid plaque (yes)	0.068 ± 0.026	3.0%	0.01	1.15 ± 0.45	2.8%	0.01
cfPWV (m/s)	0.0097 ± 0.0036	3.1%	0.008	0.24 ± 0.06	6.7%	0.0001
Hypertesion (yes)	0.050 ± 0.026	1.6%	0.054	0.90 ± 0.47	1.6%	0.054
CV disease (yes)	0.044 ± 0.042	0.5%	0.29	0.72 ± 0.72	0.4%	0.32
Diabetes (yes)	0.27 ± 0.11	2.5%	0.02	−1.46 ± 1.78	0.3%	0.41
Metabolic syndrome (yes)	0.054 ± 0.026	1.9%	0.04	0.84 ± 0.46	1.5%	0.07
BMI (kg/m^2^)	0.0051 ± 0.0033	1.0%	0.12	0.082 ± 0.063	0.7%	0.19

*Age adjusted*						
Women	−0.007 ± 0.028	0.0%	0.80	−0.49 ± 0.49	0.4%	0.32
PP (mmHg)	0.0001 ± 0.00001	0.2%	0.48	0.0002 ± 0.016	0.0%	0.86
HR (min^−1^)	0.0033 ± 0.0011	3.0%	0.007	0.047 ± 0.022	1.9%	0.04
cIMT (mm)	0.15 ± 0.13	0.5%	0.27	3.22 ± 2.41	0.7%	0.18
Carotid plaque (yes)	0.033 ± 0.028	0.6%	0.24	0.37 ± 0.48	0.2%	0.45
cfPWV (m/s)	0.0042 ± 0.0042	0.4%	0.33	0.14 ± 0.07	1.6%	0.047
Hypertesion (yes)	0.017 ± 0.027	0.2%	0.54	0.05 ± 0.50	0.0%	0.92
CV disease (yes)	0.027 ± 0.041	0.2%	0.51	0.33 ± 0.70	0.1%	0.63
Diabetes (yes)	0.26 ± 0.11	2.2%	0.02	−2.11 ± 1.70	0.6%	0.22
Metabolic syndrome (yes)	0.034 ± 0.026	0.7%	0.19	0.32 ± 0.46	0.2%	0.48
BMI (kg/m^2^)	0.0052 ± 0.0032	1.1%	0.11	0.078 ± 0.060	0.7%	0.21

*Model 1*						
Age (years)	0.0042 0.0012	4.6%	0.0009	0.069 ± 0.026	2.7%	0.01
PP (mmHg)	—	—	0.57	—	—	0.91
HR (min^−1^)	0.0033 0.0012	3.0%	0.007	0.050 ± 0.021	2.2%	0.02
cIMT (mm)	—	—	0.29	4.68 ± 2.37	1.6%	0.049
Carotid plaque (yes)	—	—	0.21	—	—	0.58
cfPWV (m/s)	—	—	0.65	—	—	0.35
Model		8.0%			12.6%	

*Model 2*						
Age (years)	0.0041 0.0012	4.3%	0.001	0.069 ± 0.026	2.7%	0.01
PP (mmHg)	—	—	0.58	—	—	0.91
HR (min^−1^)	0.0034 0.0012	3.3%	0.004	0.050 ± 0.021	2.2%	0.02
cIMT (mm)	—	—	0.34	4.68 ± 2.37	1.6%	0.049
Carotid plaque (yes)	—	—	0.31	—	—	0.58
cfPWV (m/s)	—	—	0.60	—	—	0.35
Hypertesion (yes)	—	—	0.99	—	—	0.69
CV disease (yes)	—	—	0.46	—	—	0.34
Diabetes (yes)	0.27 0.11	2.5%	0.01	—	—	0.25
Metabolic syndrome (yes)	—	—	0.43	—	—	0.42
BMI (kg/m^2^)	—	—	0.33	—	—	0.28
Model		10.6%			12.6%	

Regr coeff: regression coefficient; SE: standard error; prob level: probability level.

**Table 4:** Salivary concentrations according to anti-hypertensive treatments.

	Diuretics		Beta-blockers		Calcium blockers		ACEi or anti-AT1	
	No	Yes	No	Yes	No	Yes	No	Yes
*N*	177	64	190	51	207	34	151	90
Age	64 ± 10	70 ± 9^∗∗∗^	65 ± 10	69 ± 8^∗∗∗^	65 ± 10	70 ± 9^∗∗^	63 ± 10	70 ± 9^∗∗∗^
Women	30%	33%	30%	31%	33%	15%^∗^	32%	27%
Sodium (mM)	14.9 (12.1–19.3)	15.4 (12.9–19.2)	14.8 (12.0–17.7)	16.8 (13.7–22.0)^∗∗^	15.2 (12.3–19.45)	14.9 (11–7-17.6)	14.9 (12.0–19.8)	15.3 (12.6–17.9)
Potassium (mM)	30.3 (25.4–36.2)	34.2 (26.7–40.0)^∗^	30.1 (25.4–36.0)	34.9 (29.3–42.7)^∗∗^	31.3 (25.4–36.2)	31.2 (26.4–45.0)	31.02 (25.3–36.4)	31.7 (26.0–37.8)
Calcium (mM)	0.30 (0.24–0.39)	0.32 (0.25–0.43)	0.29 (0.24–0.39)	0.35 (0.27–0.43)^∗∗^	0.30 (0.24–0.39)	0.32 (0.26–0.49)	0.30 (0.24–0.39)	0.31 (0.25–0.42)
Phosphate (mM)	6.55 (5.22–8.92)	7.16 (5.09–9.42)	6.49 (5.09–8.82)	7.26 (5.71–9.94)	6.58 (5.13–8.77)	6.67 (5.33–11.96)^∗^	6.46 (5.13–9.05)	6.69 (5.25–8.80)

Data are expressed as either mean ± SD or median (interquartile range) for normally and nonnormally distributed data, respectively. Statistically significant differences were determined using either a Student's *t*-test (normally distributed data) or Wilcoxon signed-rank test (nonnormally distributed data) for continuous variable. A chi-square test was used for categorical data. ^∗^ *P* < 0.05; ^∗∗^ *P* < 0.01; ^∗∗^ *P* < 0.001 vs. no treatment.

## Data Availability

Data used to support the findings of this study are included within the supplementary information file.

## Supplementary Materials

Supplementary Materials Supplementary Table 1: cohort characteristics stratified according to sex. Supplementary Table 2: correlations between plasma potassium, sodium, and calcium with cardiovascular phenotypes, prevalent cardiovascular disease, and cardiovascular risk factors.
