# Supplementary material for: Differential Associations for Salivary Sodium, Potassium, Calcium, and Phosphate Levels with Carotid Intima Media Thickness, Heart Rate, and Arterial Stiffness
Source: Dis Markers. 2018 Dec 16;2018:3152146. doi: 10.1155/2018/3152146 (PMC6311732; doi:10.1155/2018/3152146)
Supplement: Supplementary Materials — Supplementary Table 1: cohort characteristics stratified according to sex. Supplementary Table 2: correlations between plasma potassium, sodium, and calcium with cardiovascular phenotypes, prevalent cardiovascular disease, and cardiovascular risk factors. [file 3152146.f1.docx]

**Supplementary Table 1: Cohort characteristics stratified according to sex.**

|  |  | | | **Men** | | **Women** | |
| --- | --- | --- | --- | --- | --- | --- | --- |
| N |  | | | 168 | | 73 | |
| Age (years) |  | | | 64 ± 10 | | 68 ± 11* | |
| Women |  | | | 0% | | 100% | |
| BMI (kg/m^2^) |  | | | 27 ± 4 | | 25 ± 4** | |
| Metabolic syndrome |  | | | 57% | | 46% | |
| Diabetes |  | | | 1.8% | | 1.4% | |
| Hypertension |  | | | 61% | | 59% | |
| CV disease |  | | | 14% | | 7% | |
| **Saliva parameters** |  | | |  | |  | |
| Sodium (mM) |  | | | 15.3 (12.4-19.8) | | 15.0 (11.7-17.7) | |
| Potassium (mM) |  | | | 31.5 (25.7-37.6) | | 30.5 (24.9-35.2) | |
| Phosphate (mM) | |  | 6.72 (5.15-9.05) | | 6.37 (5.20-8.92) | |  |
| Calcium (mM) | |  | 0.31 (0.24-0.39) | | 0.31 (0.25-0.45) | |  |
| Sodium / Potassium ratio | |  | 0.47 (0.39-0.58) | | 0.46 (0.39-0.58) | |  |
| Calcium / Phosphate ratio | |  | 0.046 (0.032-0.065) | | 0.049 (0.035-0.071) | |  |
| **Plasma parameters** |  | | |  | |  | |
| Potassium (mM) |  | | | 4.20 (4.00-4.50) | | 4.10 (4.00-4.50) | |
| Sodium (mM) |  | | | 141 (140-142) | | 142 (141-142) | |
| Calcium (mM) |  | | | 94 (93-97) | | 95 (93-98) | |
| Sodium / Potassium ratio |  | | | 33.3 (31.6-34.9) | | 34.0 (33.3-35.5) | |
| HDL Cholesterol (mg/dL) |  | | | 0.47 (0.39-0.55) | | 0.59 (0.45-0.68)*** | |
| Triglycerides (mg/dL) |  | | | 0.98 (0.75-1.44) | | 0.91 (0.74-1.14)* | |
| Glucose (g/L) |  | | | 1.00 (0.94-1.07) | | 0.98 (0.93-1.02) | |

*Data are expressed as either mean±SD or median (interquartile range) for normally and non-normally distributed data, respectively. Statistically significant differences were determined using either a Student’s t-test (normally distributed data) or Wilcoxon signed-rank test (non-normally distributed data) for continuous variable. A Chi square test was used for categorical data. *P<0.05; ***P<0.001 vs. Men*

**Supplementary Table 2: Correlations between plasma potassium, sodium, and calcium with cardiovascular phenotypes, prevalent cardiovascular disease, and cardiovascular risk factors.**

|  | **Potassium** | | | **Sodium** | | | **Calcium** | | |
| --- | --- | --- | --- | --- | --- | --- | --- | --- | --- |
|  | **Reg Coef ± SE** | **R²** | **Prob Level** | **Reg Coef ± SE** | **R²** | **Prob Level** | **Reg Coef ± SE** | **R²** | **Prob Level** |
| Age (years) | -0.001 ± 0.002 | 0.1% | 0.58 | 0.013 ± 0.015 | 0.6% | 0.37 | 0.013 ± 0.024 | 0.1% | 0.60 |
| Women (yes) | -0.042 ± 0.049 | 0.3% | 0.39 | 0.457 ± 0.329 | 1.5% | 0.17 | 0.291 ± 0.527 | 0.1% | 0.58 |
| PP (mmHg) | -0.001 ± 0.001 | 0.2% | 0.52 | 0.002 ± 0.010 | 0.0% | 0.83 | 0.027 ± 0.015 | 1.4% | 0.07 |
| HR (min^-1^) | -0.001 ± 0.002 | 0.2% | 0.49 | 0.015 ± 0.014 | 0.9% | 0.28 | 0.023 ± 0.023 | 0.4% | 0.32 |
| cIMT (mm) | -0.212 ± 0.199 | 0.5% | 0.29 | 0.949 ± 1.304 | 0.4% | 0.47 | 1.462 ± 2.132 | 0.2% | 0.49 |
| Carotid plaque (yes) | 0.013 ± 0.046 | 0.0% | 0.78 | 0.185 ± 0.298 | 0.3% | 0.54 | -0.647 ± 0.481 | 0.8% | 0.18 |
| cfPWV (m/s) | -0.003 ± 0.006 | 0.1% | 0.60 | -0.016 ± 0.044 | 0.1% | 0.71 | 0.117 ± 0.065 | 1.4% | 0.07 |
| Hypertension (yes) | -0.071 ± 0.046 | 1.0% | 0.12 | 0.012 ± 0.296 | 0.0% | 0.97 | 1.682 ± 0.480 | 4.9% | 0.0006 |
| CV disease (yes) | 0.072 ± 0.071 | 0.4% | 0.31 | 0.092 ± 0.611 | 0.0% | 0.88 | 0.159 ± 0.752 | 0.0% | 0.83 |
| Diabetes (yes) | -0.070 ± 0.176 | 0.1% | 0.69 | 0.468 ± 1.192 | 0.1% | 0.70 | -1.093 ±1.884 | 0.1% | 0.56 |
| Metabolic syndrome (y) | 0.044 ± 0.045 | 0.4% | 0.33 | 0.056 ± 0.298 | 0.0% | 0.85 | 0.980 ± 0.481 | 1.7% | 0.04 |
| BMI (kg/m²) | -0.002 ± 0.006 | 0.1% | 0.70 | 0.001 ± 0.041 | 0.0% | 0.99 | 0.150 ± 0.061 | 2.5% | 0.01 |

*Regr coeff: regression coefficient; SE: standard error; Prob level : probability level*
